# Effects of Regular Exercise and Intermittent Fasting on Neurotransmitters, Inflammation, Oxidative Stress, and Brain-Derived Neurotrophic Factor in Cortex of Ovariectomized Rats

**DOI:** 10.3390/nu15194270

**Published:** 2023-10-06

**Authors:** Tarfa Albrahim, Raghad Alangry, Raghad Alotaibi, Leen Almandil, Sara Alburikan

**Affiliations:** Department of Health Sciences, Clinical Nutrition, College of Health and Rehabilitation Sciences, Princess Nourah Bint Abdulrahman University, Riyadh 11671, Saudi Arabia; 441001678@pnu.edu.sa (R.A.); 441000174@pnu.edu.sa (R.A.); 441001461@pnu.edu.sa (L.A.); 441004769@pnu.edu.sa (S.A.)

**Keywords:** ovariectomy, regular exercise, intermittent fasting, neuroinflammation, oxidative stress, BDNF

## Abstract

A collection of metabolic disorders and neurodegenerative diseases linked to oxidative stress and neuroinflammation frequently affect postmenopausal women or estrogen deprivation. Recent research has focused on alternative therapies that can enhance these women’s quality of life. This study set out to investigate the effects of physical exercise (EX) and intermittent fasting (IF) on oxidants/antioxidants, inflammatory cytokines, neurotransmitters, and brain-derived neurotrophic factor (BDNF) in the cortex of rats. Additionally, it sought to assess the response to oxidative stress and neuroinflammation in the brains of rats following ovariectomy (OVX) and the potential mechanisms of these interventions. Fifty female rats were divided into one of the following groups 30 days after bilateral OVX: Control, OVX, OVX + EX, OVX + IF, and OVX + EX + IF groups. The rats in the Control and OVX groups continued their normal activities and had unrestricted access to food and water, but the rats in the OVX + EX and OVX + EX + IF groups had a 4-week treadmill training program, and the rats in the OXV + IF and OVX + EX + IF groups fasted for 13 h each day. The rats were killed, the cerebral cortex was taken, tissue homogenates were created, and various parameters were estimated using these homogenates. The results show that ovariectomized rats had decreased levels of neurotransmitters (DA, NE, and SE), acetylcholinesterase, brain GSH (glutathione), SOD (superoxide dismutase), catalase, GPx (glutathione peroxidase), and TAC (total antioxidant capacity), as well as elevated levels of proinflammatory cytokines and mediators (TNF-α, IL-1β, Cox-2). While ovariectomy-induced declines in neurotransmitters, enzymatic and nonenzymatic molecules, neuroinflammation, and oxidative brain damage were considerably mitigated and prevented by treadmill exercise and intermittent fasting, BDNF was significantly increased. These results suggest that ovariectomy can impair rat neuronal function and regular treadmill exercise and intermittent fasting seem to protect against ovariectomy-induced neuronal impairment through the inhibition of oxidative stress and neuroinflammation and increased BDNF levels in the brain cortex. However, combining regular exercise and intermittent fasting did not provide additional benefits compared to either treatment alone.

## 1. Introduction

Numerous disorders, including osteoporosis, cardiovascular disease, chronic kidney disease (CKD), and dementia, are largely caused by oxidative stress and inflammation. Free radicals have an impact on a variety of enzymes, cellular signaling proteins, and the structure of cell membranes [1]. The synthesis of lipid peroxides and proinflammatory chemicals is found to increase during menopause, whether it be physiological or surgical [2], which causes an estrogen deficiency and the aetiology of various alterations that frequently affect menopausal women. Following ovarian surgery, the prevalence of cardiovascular and renal issues increases while estrogen levels decrease [3]. Another crucial role for estrogen is the main control of energy homeostasis [4]. Estrogen influences a variety of biological functions through its receptors [5]. There is growing proof that the postmenopausal state induces metabolic alterations and oxidative imbalance that contribute to the aetiology of a number of diseases. According to the research of Ishikawa et al. [2], the reduction in estrogen during menopause is followed by an increase in oxidative stress. Recent experimental research of Yalin et al. [6] has shown that ovarian removal surgery (OVX) results in oxidative stress in mice. Furthermore, the earlier scientific research validated this idea by assessing different oxidative stress markers in postmenopausal women [5]. Additionally, multiple studies have demonstrated that a reduction in estrogen production causes histopathological changes and an infiltration of inflammatory cells [7]. In general, brain disorders are tightly associated with inflammatory and oxidative stress responses. TNF-alpha and IL-1, two cytokines that have the capacity to start inflammatory cascades, may have a deleterious effect on neuronal function [8]. Therefore, estrogen loss is responsible for the imbalance between increased reactive oxygen species (ROS) production and decreased antioxidant activity. As a result, the incidence of Alzheimer’s disease (AD) in postmenopausal women is also nearly three times greater than in men their age, according to a large body of data [9]. A woman’s elevated risk of AD is strongly connected with a decline in estrogen levels during the various menopausal phases. In addition to preventing the development of Aβ and its toxicity, improving neurotrophy and promoting the growth and survival of memory-associated cholinergic neurons are both possible effects of estrogen [10].

Physical inactivity has been found to have several disadvantages for one’s health [11,12], making it the fourth leading cause of mortality in the world and one of the most important public health concerns of the twenty-first century [13]. Exercise can have both positive and negative physiological consequences depending on its intensity [14]. According to encouraging evidence from basic science and clinical investigations, physical exercise is being used as a treatment for a number of prevalent neurological and psychiatric illnesses (CNS). This is facilitated by an increase in neurotrophic factors, such as brain-derived neurotrophic factor (BDNF) [15,16], a reduction in oxidative stress [17], and a limitation of neuroinflammation [18]. However, the exact mechanisms through which exercise influences the brain are still not fully understood. By boosting mitochondrial oxygen consumption, exhausting exercise, on the one hand, promotes the production of reactive oxygen species (ROS), which can enhance oxidative stress and increase oxidative damage to lipids, proteins, and DNA [19]. In addition, multiple investigations have shown that superoxide is secreted into the extracellular space during muscular contraction. Upon swiftly entering the bloodstream, this superoxide interacts with other cells to encourage membrane lipid peroxidation, protein denaturation, and a number of cell signaling pathways, which in turn sets off inflammatory and cell death pathways [20]. Regular exercise, on the other hand, causes an adaptation of the antioxidant system response by increasing the tolerance to oxidative stress [14]. Many cells react to exercise-induced increases in ROS and oxidative stress by using endogenous defense mechanisms, such as adjustments in enzymatic and nonenzymatic endogenous antioxidants.

Intermittent fasting (IF) has been acknowledged as one of the dietary restriction (DR) treatments that has been shown to have anti-inflammatory and antioxidant properties in both people and animals [21], even though its underlying molecular mechanisms are still unknown [22]. However, IF has been proven to increase glucose tolerance, lower blood cholesterol, and lower fasted insulin levels [23]. In the past ten years, mitochondrial dysfunction has become a key pathomechanism of cardiometabolic diseases. Calorie restriction (CR) is a key factor in the normalization of mitochondrial quality and quantity in illness. However, the precise processes underlying the improvement in mitochondrial function and dynamics are not well understood [24]. Several investigations have shown that CR and IF may develop the protective properties of mitochondria. In this regard, CR increased mitochondrial fission and fusion proteins and attenuated the age-related mitochondrial fragmentation [25,26]. Furthermore, numerous studies have shown the beneficial effects of CR and IF on mitochondria-related ROS production. Long-term exposure to CR has been shown to significantly reduce (by 30%) both oxidative stress and mitochondrial DNA damage. Through the lowering of ROS formation, even brief (3 weeks) episodes of CR were able to stop the progression of isoproterenol-induced heart hypertrophy. Rats subjected to a 24-month alternate-day fasting protocol showed reduced oxidative stress (measured by elevated glutathione levels) and a decreased inflammatory state in the heart, similar to that of young animals [27]. Along with optimizing the metabolic pathways, every-other-day IF in conjunction with a high-intensity intermittent exercise routine resulted in significantly lower MDA levels and lower plasma protein oxidation [28]. The impact of CR and IF on antioxidant mitochondrial enzymes as opposed to cytosolic enzymes is an intriguing finding. It was discovered that two months of CR dramatically boosted mitochondrial (but not cytosolic) SOD and GPx activities in rats [29].

To the best of our knowledge, a model of an ovariectomized rat has not previously been used to explore the effects of IF and/or exercise on redox status, neuroinflammation, and several neurological parameters.

## 2. Materials and Methods

### 2.1. Animal Preparation

Fifty female Sprague Dawley rats, 8 weeks old and weighing about 300 ± 20 g each, were kept in the animal experimentation and surgical lab at King Saud University’s faculty of medicine. All the animals were kept in environments that were kept at a constant 22.1 °C temperature, 50–55% humidity, and a 12 h day/night cycle. All experimental procedures were by the guidelines of the Institutional Animal Care and Use Committee (IACUC) of Princess Nourah bint Abdulrahman University (approval no HAP-01-R-059; IRB Log Number: 22-1141).

### 2.2. Experiment Design

All the rats were allocated into five groups of ten at random, as follows: Control group, OVX group, OVX with exercise (EX), OVX with intermittent fasting (IF), and OVX with both exercise and IF groups.

### 2.3. Bilateral Ovariectomy Procedure

In a rat chamber, all animals underwent thorough anesthesia utilizing flows of 8% sevoflurane inhalation anesthesia and 3% oxygen. The rats were kept in the chamber until they were completely unconscious. The rat was placed on a surgical table with a constant temperature of 37 °C. The muscle was then delicately opened after 1 cm of the skin in the center of the pelvis was carefully opened with a sterilized fine surgical blade. Then, using locking straight forceps secured to the tube and the ligament, both ovaries were gently pushed out. A surgical cautery device was then used to remove the ovary while minimizing bleeding. Following that, two sutures were used to seal the muscle and skin. Following surgery, the recuperation area was used for all rats under veterinary care until they healed. After that, all animals were returned to their cages.

### 2.4. Exercise Session

Postmenopausal female rats in the chronic phase were trained on the treadmill for eight sessions spread over two weeks following a month of bilateral ovariectomy. Then, the animals were subjected to exercise four times per week for 15 min at 18–25 m/s on a 0% slope.

### 2.5. Intermitting Fasting

After one month of bilateral ovariectomy, postmenopausal female rats were starved from dusk to dawn in Riyadh time, which equates to roughly 13 h. By taking away the food and water at dusk and bringing them back at dawn, the fasting was made mandatory.

### 2.6. Sampling and Sacrificing

Ketamine/xylazine was administered to all animals at 9.1/91 mg/kg to induce anesthesia. The cerebral cortex was detached right away and given an isotonic saline wash. The dissected specimens were divided into three parts, and one part was quickly frozen at −80 °C for RNA extraction. For biochemical analyses, the second cerebral cortex tissue part was homogenized to a 10% (*w*/*v*) homogenate in ice-cold 10 mM phosphate buffer (pH 7.4). At the same time, the third part was homogenized in 75% HPLC grade methanol (10% *w*/*v*) and centrifuged for 10 min. at 4000 rpm to assess monoamine levels in the supernatant.

### 2.7. Estimation of Redox Status

#### 2.7.1. Oxidative Stress Index Evaluation

Using the thiobarbituric acid method established by Ohkawa et al. [30], malondialdehyde (MDA) was measured to reflect the degree of lipid peroxidation. As previously stated [31], dye production after the injection of Griess reagent was detected at 540 nm to determine the quantity of nitric oxide (NO) in the cerebral cortex. As previously mentioned [32], the yellow chromogen was determined at 412 nm and GSH levels were calculated using Ellman’s reagent. Furthermore, to assess total antioxidant capacity (TAC), an Abcam (Catalog Number: ab65329; Cambridge, UK) colorimetric assay kit was used according to the manufacturer’s instructions.

#### 2.7.2. Antioxidant Activity Assessment

According to the methods outlined by Paglia and Valentine [33], glutathione peroxidase (GPx) was evaluated. Using the approach outlined by Aebi [34], the activity of the catalase (CAT) enzyme in the cortical region was calculated. In addition, 480 nm measurements of cortical total superoxide dismutase (TSOD) activity were made using the method outlined by Misra and Fridovich [35]. To measure SOD2 activity, the method of de Haan et al. [36] was employed. The subtraction of the activity remaining after the addition of potassium cyanide (SOD2) from total SOD activity yielded SOD1 activity.

#### 2.7.3. Determination of Inflammatory Cytokines

For the determination of cortical inflammatory cytokine levels, cyclooxygenase-2 (COX-2), interleukin 1 beta (IL-1β), and tumor necrosis factor alpha (TNF-α) ELISA kits (MyBioSource, San Diego, CA, USA) were used according to the manufacturer’s instructions (Catalog Numbers: MBS725633, MBS2023030, and MBS175904, respectively).

#### 2.7.4. Estimation of BDNF Protein

According to the manufacturer’s instructions, cortical brain-derived neurotrophic factor (BDNF) was assayed using ELISA kits sourced from Abcam (Catalog Number: ab21389; Cambridge, UK).

#### 2.7.5. Assessment of Acetylcholinesterase and Monoamines

According to the method outlined by Elman et al. [37], the cortical activity of acetylcholinesterase (AChE) was evaluated. Thionitrobenzoic acid was added, and the yellow color that resulted was detected at 412 nm to evaluate AChE activity. Using the data acquisition program (ChemStation), HPLC reports and chromatograms were obtained. To remove trace elements and lipids from cerebral cortex samples, a solid-phase extraction was performed using a CHROMABOND column (Cat. No. 730031), and the NH2 phase was then obtained. The NH2 phase was then injected into a 150 mm by 5 µm by C18 Phenomenex, USA, AQUA column. Dopamine (DA), norepinephrine (NE), and serotonin (5-HT) were isolated after 12 min. By comparing the obtained chromatogram with that of the appropriate standard (Sigma Chemical Co., St. Louis, MO, USA), it was possible to determine the position and concentration of each monoamine in the sample. The amount of each monoamine in relation to the total amount of brain tissue was measured, as per Pagel et al. [38].

#### 2.7.6. Real-Time Quantitative Polymerase Chain Reaction

With the support of the TRIzol reagent (Invitrogen, Life Technologies Corporation, Carlsbad, CA, USA), the total cellular RNA was isolated from the cortical tissues. The reverse transcription reaction solution was then made by combining 1 µL total cellular RNA with 4 µL Prime Script TM Master Mix (Takara Biomedical Technology Beijing Co., Ltd., Beijing, China) and 15 µL RNase-free ddH_2_O. The exact circumstances were 37 °C for 15 min, 85 °C for 5 s, and 4 °C for 10 min. In order to create the mixed solution for the Q-PCR experiment, 5 µL of SYBR Green Mix (Bio-Rad, Hercules, CA, USA), 2 µL of cDNA template, and upstream and downstream primers were added. These conditions were 1 cycle at 95 °C for 5 s, followed by 40 cycles at 95 °C for 5 s and at 60 °C for 31 s. The following are the melting conditions: 60 °C for 30 s, 95 °C for 15 s, and 60 °C for 15 s. Finally, using the 2(−ΔΔCt) technique, the relative fold change was determined. The primers were synthesized by Sigma-Aldrich (St. Louis, MO, USA). The sequences are displayed in Table 1.

### 2.8. Statistical Analysis

The standard deviation (SD) and the mean are used to express data. Using the statistical program SPSS version 23.0, measurements were analyzed using one-way analysis of variance (ANOVA), followed by a Duncan’s post hoc test. *p*-values less than 0.05 were regarded as significant.

## 3. Results

**Figure 1:**
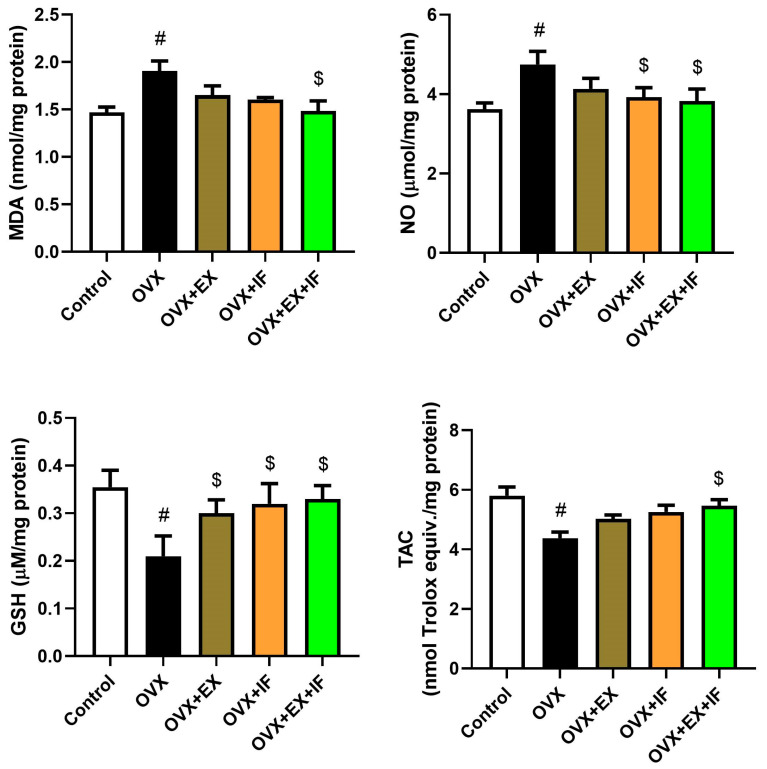
Effects of exercise four times per week and intermittent fasting 13 h/day for 4 weeks on oxidant/antioxidant (MDA, NO, GSH, and TAC) levels in cortical tissue of ovariectomized female rats. The results are presented as mean ± SD values (*n* = 10). The letters (^#^ and ^$^) indicate statistical differences at *p* < 0.05 in comparison with the control and OVX groups, respectively.

**Figure 2:**
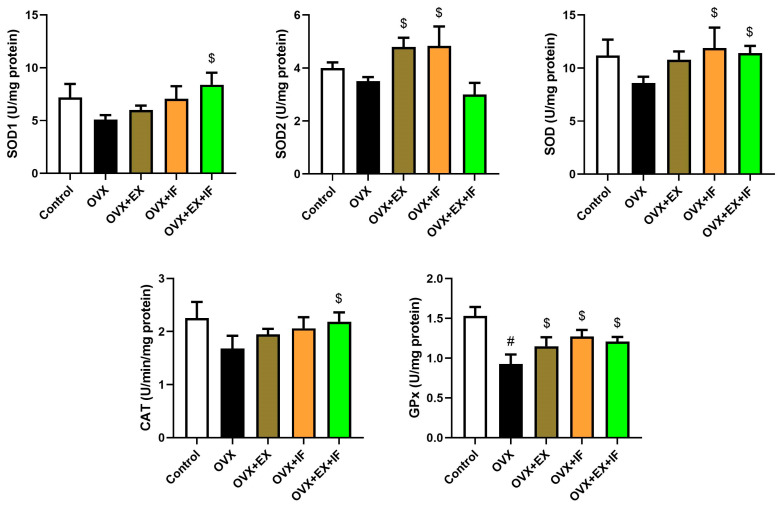
Effects of exercise four times per week and intermittent fasting 13 h/day for 4 weeks on antioxidant enzymes (SOD1, SOD2, TSOD, CAT, and GPx) activities in cortical tissue of ovariectomized female rats. The results are presented as mean ± SD values (*n* = 10). The letters (^#^ and ^$^) indicate statistical differences at *p* < 0.05 in comparison with the control and OVX groups, respectively.

**Figure 3:**
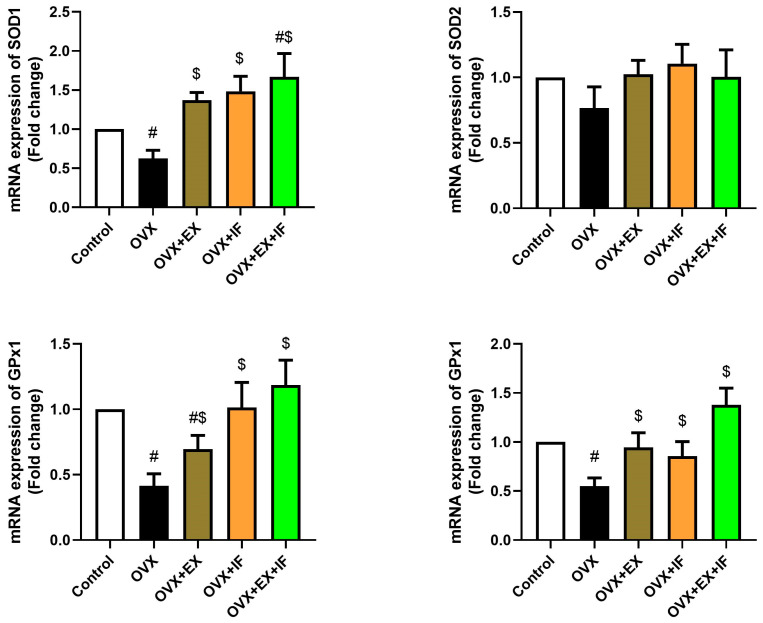
Effects of exercise four times per week and intermittent fasting 13 h/day for 4 weeks on mRNA expression levels of superoxide dismutase and glutathione peroxidase in cortical tissue of ovariectomized female rats. The results are presented as mean ± SD (*n* = 6) of triplicate assays and adjusted to GAPDH. The letters (^#^ and ^$^) indicate statistical differences at *p* < 0.05 in comparison with the control and OVX groups, respectively.

Ovariectomy in rats induced oxidative stress evidenced by a significant increase in MDA and NO concurrently with a marked decrease in GSH (Figure 1) associated with a significant inhibition in antioxidant enzymes, namely, GPx, whereas SOD and CAT were decreased nonsignificantly (Figure 2). Oxidant/antioxidant imbalance in ovariectomized rats’ brains was confirmed by a significant reduction in the total antioxidant capacity (TAC) level (Figure 1). Interestingly, exercise was effective in the restoration of oxidant/antioxidant balance in ovariectomized rats where the oxidative stress markers were nonsignificantly increased compared to the control rats. Fasting of ovariectomized rats significantly attenuated the oxidative stress induction. Moreover, combination of fasting and exercise was effective in the restoration of the redox status in ovariectomized rats rather than the use of EX or IF alone. However, the combination of EX and IF together was not able to enhance SOD2 activity in the cortical tissue of ovariectomized rats. mRNA expression of SOD1, SOD2, GPx1, and GPx4 was confirmed by the biochemical results as indicated by significant downregulations in SOD1, GPx1, and GPx4 and a nonsignificant downregulation in SOD2 in ovariectomized rats, and fasting successfully attenuated this downregulation (Figure 3).

**Figure 4:**
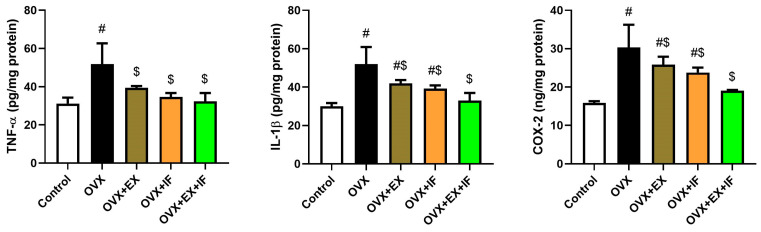
Effects of exercise four times per week and intermittent fasting 13 h/day for 4 weeks on proinflammatory cytokine/mediator (TNF-α, IL-1β, and Cox-2) levels in cortical tissue of ovariectomized female rats. The results are presented as mean ± SD values (*n* = 10). The letters (^#^ and ^$^) indicate statistical differences at *p* < 0.05 in comparison with the control and OVX groups, respectively.

Ovariectomy significantly increased the proinflammatory cytokines/mediators compared to the control rats (*p* < 0.05). The exercise fortunately prevented the inflammatory response in ovariectomized rats (Figure 4). Moreover, the fasting of the ovariectomized rats reduced the inflammatory response evidenced by the significant reduction in TNF-α, IL-1β, and Cox-2. Furthermore, the combination of fasting and exercise successfully restrained proinflammatory cytokine (TNF-α and IL-1β) and mediator (Cox-2) elevations.

**Figure 5:**
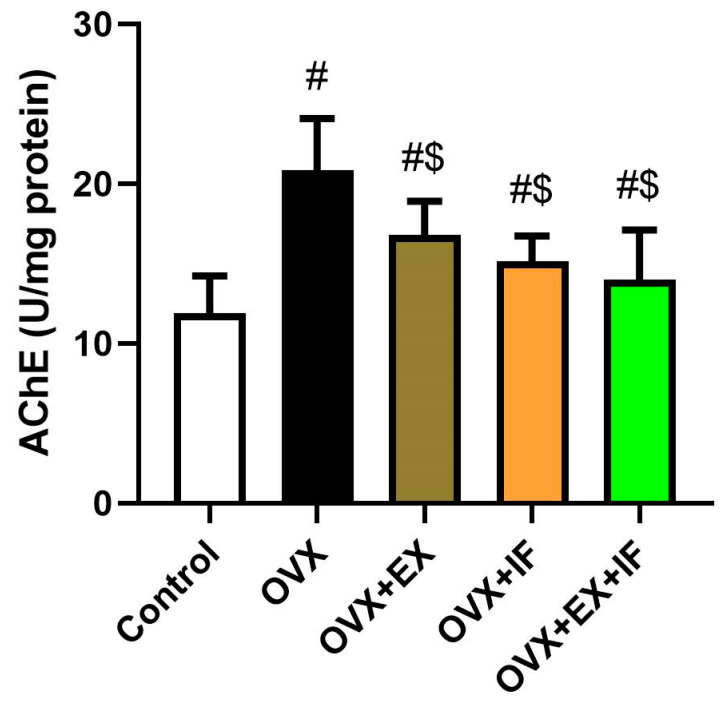
Effects of exercise four times per week and intermittent fasting 13 h/day for 4 weeks on acetylcholinesterase activity in cortical tissue of ovariectomized female rats. The results are presented as mean ± SD values (*n* = 10). The letters (^#^ and ^$^) indicate statistical differences at *p* < 0.05 in comparison with the control and OVX groups, respectively.

**Figure 6:**
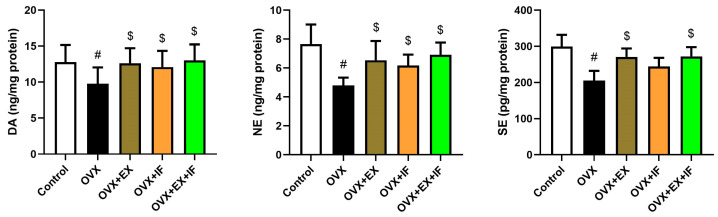
Effects of exercise four times per week and intermittent fasting 13 h/day for 4 weeks on neurotransmitters (DA, NE, and SE) level in cortical tissue of ovariectomized female rats. The results are presented as mean ± SD values (*n* = 10). The letters (^#^ and ^$^) indicate statistical differences at *p* < 0.05 in comparison with the control and OVX groups, respectively.

Acetylcholinesterase (AChE) activity in the cortex of ovariectomized rats was significantly increased (Figure 5), whereas fasting and exercise, each one alone, were able to restrain it and returned its activity to the control values. Interestingly, both fasting and exercise were able to inhibit AChE activity and restored it near to the control levels; however, the activity of AChE was still significantly higher than the control values. Furthermore, monoamines, namely, dopamine, norepinephrine, and serotonin, were declined significantly in the cerebral cortex of ovariectomized rats (Figure 6). However, the use of regular exercise or intermittent fasting application alone was able to restore their levels to the control values. Unfortunately, the combination of EX and IF did not show a significant effect compared with OVX + EX and OVX + IF, indicating the combination was not effective.

**Figure 7:**
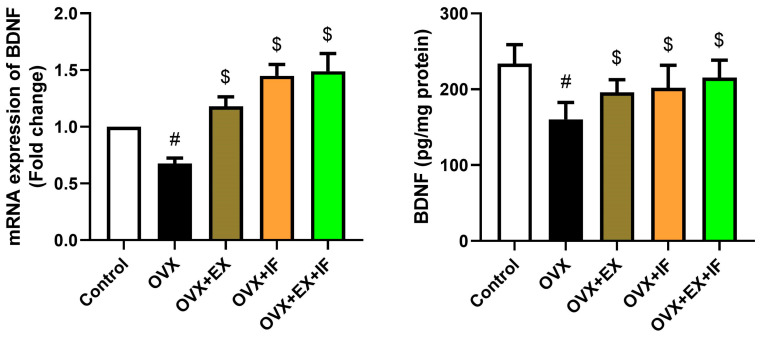
Effects of exercise four times per week and intermittent fasting 13 h/day for 4 weeks on brain-derived neurotrophic factor (BDNF) mRNA expression and level in cortical tissue of ovariectomized female rats. The biochemical results are presented as mean ± SD values (*n* = 10), while the mRNA expression results are presented as mean ± SD (*n* = 6) of triplicate assays and adjusted to GAPDH. The letters (^#^ and ^$^) indicate statistical differences at *p* < 0.05 in comparison with the control and OVX groups, respectively.

The brain BDNF mRNA expression and level were decreased significantly in ovariectomized rats compared to control rats (Figure 7). However, exercise alone with ovariectomy was successful to increase BDNF. Moreover, fasting alone or concurrently with exercise in ovariectomy rats significantly enhanced the level of BDNF compared to OVX rats, but the level was still significantly lower when compared to control rats.

## 4. Discussion

According to Ge et al. [39], perimenopausal emotional disorder is a category of mood disturbed mental illness that first manifests during the perimenopausal stage and is characterized by emotional disorder, thinking impairment, and cognitive decline. In addition, a number of studies showed that most mammals experienced an antioxidative/oxidative imbalance after having an ovariectomy or an ovariohysterectomy that caused an estrogen deficit [40]. Gunay et al. [41] claimed that after OVX in heifers the levels of oxidative stress indicators rose. The results of the current investigation revealed that in OVX rats MDA and NO concentrations in the brain considerably rose while TSOD and GPx activity decreased. These findings are in line with earlier research demonstrating that ovariectomy alters the redox profile, increases oxidative stress, and subsequently speeds up the cellular ageing process in several tissues [42,43]. Since there is evidence in the literature of estrogen’s protective role, either through the activation of antioxidant enzyme genes or through direct chemical action of estrogen in removing free radicals [44], our results can be explained by the loss of the estrogen sex hormone in the OVX group. Additionally, Gomez-Zubeldia et al. [45] discovered that rat uterine MDA concentrations were lower and SOD activity was somewhat higher at day 15 following ovariectomy. However, a different study found that the TSOD and GPx activities in rats on day 30 following ovariectomy were unaffected [46]. In this investigation, group I showed higher MDA concentrations and lower GPx activity than did group II; however, these differences were not statistically significant by day 10 (*p* > 0.05). This condition was thought to be caused by oxidative stress. MDA inhibits the formation of nucleic acids and proteins as well as deactivates enzymes since it has a strong affinity for amino groups [1]. As a result, increased lipid peroxidation may be the cause of the decline in femur antioxidant enzymes seen following OVX. According to several studies [47,48], estrogen deficiency during menopause is actually linked to increased oxidative stress because of an imbalance between the synthesis and elimination of reactive oxygen species through the antioxidant response system [49]. Additionally, ovariectomy has been shown to worsen oxidative stress through altering the brain’s redox profile [50]. Indeed, estrogen deficiency is tightly linked to metabolic dysfunction and cellular redox imbalance through fat accumulation, insulin resistance, and altered mitochondrial function [51].

Enzymes that fight free radical-induced tissue or cellular damage are known as antioxidants. Either free radicals or reactive oxygen intermediates are metabolized by them to nonradical compounds. A family of glutathione-dependent enzymes is among these enzymes [52]. According to this study, rats that had their ovaries removed had significantly lower levels of the antioxidant enzymes SOD, GPX, and GST in their femurs. These results imply that the decreased female reproductive function caused oxidative damage and oxidant/antioxidant imbalance. Catalase’s capacity to avert osteopenia in mice with ovariectomies highlights the significance of antioxidants for bone health [53]. The suppression of osteoclastic development by GPx overexpression in RAW cells [53] and a decline in GPx levels in the plasma of patients with osteopenic patients [54] further highlight the potential significance of our findings.

Hormone therapy can prevent metabolic abnormalities that menopausal women are prone to developing and fat accumulation [55]. However, such treatment raises the risk for cancer and cardiovascular diseases in addition to other issues (such as stroke, pulmonary embolism, dementia in ageing, gall bladder disease, and urine incontinence), suggesting the need for novel strategies to lessen the impact [56]. Thankfully, many menopause-related symptoms can be alleviated by increasing physical activity [55]. The American Heart Association and the American College of Sports Medicine both advise older women to engage in exercise because it is a low-cost intervention that has numerous advantages for women’s health after menopause [57]. In their study of postmenopausal overweight/obese women’s whole-body fat oxidation during acute treadmill activity, Stein and colleagues [58] discovered an antagonistic association between metabolic health and the capacity to burn fat in response to aerobic exercise. This shows that higher metabolic rigidity is linked to increased lipid accumulation, which results in increased body fat and visceral fat distribution [59]. As a result, the clinic may benefit from the findings from OVX animals given here. Our findings suggest that multiple routes may be used by exercise training to protect the OVX rat brain from oxidative damage. For instance, although the CAT activity was higher in the group receiving endurance exercise training, the SOD activity was higher in the group receiving E2 replacement therapy. The only OVX group with a higher total antioxidant capacity (FRAP) in comparison to the other OVX groups was endurance exercise training. Our findings concur with those of Silva et al. [60], who discovered that exercise training enhanced the antioxidant defense system on the skeletal muscle of rats with ovariectomies.

Additionally, this result is in line with a prior experiment conducted by Utami Mulyaningrum et al. [61], which suggested that Dawood fasting (DF) might be used as a substitute for dietary restriction in order to raise SOD, GPx, and Cat antioxidant enzyme levels. This implies a DF increase in TAC levels. Furthermore, this research supports a prior study by Carlos Vinicius Dalto da Rosa et al. (2018). As a result of the food restriction protocol (FR), which called for obtaining only 50% of the average food intake of the control group—which served as the baseline for all groups—they observed that the antioxidant capacity was improved in the plasma of the DER (Diabetic with Food Restriction) group. Furthermore, considering that intermittent fasting is a more stringent measure than food restriction and that healthy rats may have higher levels of total antioxidant capacity (TAC) than diabetic rats, their results also showed a slight increase in the TAC for the CCR (control with food restriction) group. It is anticipated that calorie restriction and the management of the amount of calories entering the body will inhibit and control oxidative stress in tissues. As a result, the body produces less MDA at lower quantities [62]. The rats who were subjected to intermittent fasting had a lower TAC than groups that were not, according to the results of the current study. Furthermore, Françoise Wilhelmi de Toledo et al. [63], who concur with the current study, report that 109 people have successfully completed a 10-day fasting protocol, which resulted in weight loss, improved cardiovascular parameters, decreased lipid peroxidation, and an elevated plasma antioxidant capacity. By using a strategy that divided the participants into three groups according to their prefasting GSH values, the researchers discovered that all three groups’ lipid peroxidation and total antioxidant capacity were affected by the fasting regimen in the same way.

A class of steroidal hormones with neuroregulating and neuroprotective properties is called ovarian hormone, or the natural neuroprotective factor. Studies indicate that estrogen may have an impact on neural conduction, synapse formation, neuronal spine density, neuron proliferation, and neurotransmitter synthesis. Furthermore, it suppresses neuron death, encourages the development of nerve fibers, and controls the growth of astrocytes and microglial cells [64,65]. Simultaneously, estrogen receptors are found in many areas linked to anxiety and depression. Through the estrogen receptor, estrogen can penetrate the blood–brain barrier and influence a variety of emotion-associated neurotransmitter systems; for example, norepinephrine, dopamine, and 5-hydroxytryptamine are effective in treating emotional and mental disorders [66]. Consequently, it influences emotional responses and cognitive function, controls the structure and function of the central nervous system (CNS), and has a neuroprotective impact. Therefore, the most common treatment for perimenopausal emotional illness in clinics nowadays is estrogen replacement therapy (ERT) or estrogen plus an antidepressant [39].

Reductions in progesterone and estrogen levels during surgical or natural menopause lead to an imbalance in neurochemical brain communication, which in turn impacts neurotransmitter pathways. Studies have shown increases in noradrenaline and cortisol [67,68] and decreases in dopamine, serotonin, androstenedione, testosterone, estradiol, GABA, β-endorphin, and allopregnanolone, among other neurotransmitter pathways [69]. According to Monteleone et al. [69], all of these alterations result in neuroanatomical alterations in brain regions related to anxiety and depression, including the raphe nucleus, hippocampus, and cerebral cortex. Moreover, it is believed that decreases in estrogens after ovariectomy affect cognitive functions partly through diminishing the activity of cholinergic afferents to the hippocampus [70,71], but decreased cholinergic input to the entorhinal cortex is also probably going to have an effect. We have discovered that ovariectomy causes decreases in cortical acetylcholinesterase (AChE). Our findings are consistent with those of Batallán Burrowes et al. [70], who discovered that OVX decreased the levels of M1 and AChE receptor protein in the medial and lateral entorhinal cortex and that continuous replenishment of 17β-estradiol mitigated these reductions. Moreover, the disruption of central cholinergic neuronal pathways in the basal forebrain is strongly linked to the estrogen deficiency that led to Aβ accumulation. Furthermore, the data indicated a disruption in the neurotransmission systems of the NE, SE, and DA. This disruption may be brought on by the constant influx of Aβ as a result of estrogen shortages [72]. The rise in these molecules’ turnover rates in the brain could be the cause of catecholamine depletion [73]. Nonetheless, OVX rats benefit from exercise. It has been demonstrated that regular physical exercise has therapeutic benefits [74], including the treatment of mental illnesses [75], aiding in the recovery from brain injuries [76], and fending against neurodegenerative diseases [77]. Among these effects, the exercise-induced neural adaptation has been connected to the production of neurotransmitters, particularly monoamines. The “Central Fatigue Hypothesis,” which indicated that elevated brain 5-HT release was connected to central weariness, served as the original inspiration for the relationship between exercise and monoamines [74]. The midbrain, striatum, hypothalamus, and hippocampus of rats that undergo intense treadmill activity have increased 5-HT levels [78]. While DOPAC, the primary DA metabolite, has higher levels in both the midbrain and the striatum, dopamine levels are only elevated in the striatum. Conversely, prolonged treadmill use lowers NE levels in the brain stem and hypothalamus [79]. Additionally, brain homogenates of rats given eight weeks of food-reinforced running-wheel exercise show increased concentrations of dopamine (DA), accompanied by a corresponding downregulation of DA receptor densities in these animals [80]. Additionally, exercise shields the DA neuron from toxic insults [74]. Furthermore, in comparison to inactive individuals, persistent treadmill and wheel exercise both raise levels of NE in the pons-medulla and spinal cord [81]. Similar to this, prolonged treadmill activity raises levels of NE in brain areas related to cognitive function, such as the hippocampus and the central and medial amygdala [82]. Furthermore, 5-HT, dopamine, and glutamic acid levels were regulated by IF in both diabetic and normal rats. Ad libitum and T2DM rats’ 5-HT dramatically increased in response to fasting. Prior research has demonstrated that calorie restriction and fasting alleviate depressive symptoms [83]. Other investigations using rodent models demonstrated that fasting increased the obtainability of serotonin and tryptophan in the brain [84], which is consistent with our results. Moreover, 5-HT is reabsorbed from the synaptic cleft by the serotonin reuptake transporter (5-HTT) and stored in vesicles, where it is either deactivated by monoamine oxidase (MAO) enzymes or stored. Rats’ frontal cortex 5-HTT density was significantly reduced after two weeks of 50% food restriction, leading to an increase in 5-HT levels [85]. In the current investigation, the 5-HT level was significantly lower in OVX rats than in control rats.

Our findings showed that there was a substantial difference in serotonin levels between the brains of fasting and nonfasting subjects. According to Fuenmayorl and Garcia [86], fasting increases the 5-HT turnover in the brain and raises the possibility that this phenomenon occurs in all parts of the brain. Furthermore, they claimed that there appears to be a concomitant increase in amine release from hypothalamic neurons with this elevated turnover. Short-term fasting has been shown to raise extracellular 5-hydroxyin-dole-3-acetic acid (5-HIAA), and in the fasting group tryptophan, a precursor amino acid of 5-HT, was shown to be more concentrated in the blood. These findings were made by Japanese researchers [84]. Research from other studies has also demonstrated that eating less raises serotonin levels. According to research by Jeong Won Jahng et al. [87], a five-week dietary restriction in a rat model significantly raised serotonin levels in the hypothalamus while decreasing serotonin turnover 5-HIAA/5-HT. However, our investigation yielded varied outcomes from several studies. For instance, it was discovered that both tryptophan, a precursor to serotonin, and serotonin levels significantly dropped in a study conducted to look into the variations in serotonin metabolism and sex differences in alterations of this neurotransmitter in 4-week chow-restricted rats. The rats underwent a 4-week dietary restriction, resulting in a 20–25% drop in body weight [88]. The type of dietary restrictions appears to be the cause of the discrepancy between the results of the latter study and the current study. Long-term dietary limitations are thought to have the potential to deplete tryptophan reserves, which could then result in a drop in serotonin levels. However, tryptophan levels in the current study were not lowered due to the nourishing meal. Furthermore, the body weight of the feeding-limited rats in the aforementioned study was considerably lower than that of the controls, suggesting that the food was overly restricted. In this investigation, no appreciable variations in the plasma levels of dopamine between the fasting subjects and the control group were noted. According to research by Rosberry [89], fasting or dietary restriction may alter the characteristics of somatodendritic dopamine release by raising dopamine release. According to a recent review of research on the effects of fasting on mood, persons who undergo modified fasting for seven days experience an increase in dopamine and other neurotransmitter plasma levels [90]. Bastani et al. [91], however, reported that brief fasting may have an impact on monoamine metabolism, which in turn lowers the levels of extracellular striatal dopamine in young rats.

OXV rats in this study displayed signs of neuroinflammation, as shown by an increase in proinflammatory mediators and cytokines. The acquired results are consistent with the findings of Ge et al. [39], who discovered that proinflammatory factors were elevated and M1 polarization activation of microglial cells was encouraged by estrogen deprivation. According to the research, OVX significantly increased the levels of proinflammatory cytokines (IL-1β, IL-6, and TNF-α) and oxidative stress factor (iNOS) in the prefrontal cortices and exacerbated microglial cell activation and M1 polarization. Its upstream mechanism may involve triggering M1 polarization and microglial cell activation (downregulation of Arg1 and overexpression of iNOS). Moreover, the proinflammatory action of NF-κB on microglial cells is indirectly mediated by activating microglial cells in response to chronic stress [49]. This could be another explanation for the inflammation. The activation of NF-κB is linked to stress-induced neuronal damage as it stimulates the manufacture of proinflammatory cytokines such as TNF-α and activates the expression of iNOS in the prefrontal cortex [39,92].

Exercise, however, was able to reduce this type of neuroinflammation. Accordingly, prior research has shown that, in order to achieve a neuroprotective impact, two or three weeks of preischemia exercise are required [93,94]. Furthermore, preconditioning exercise has been shown in a number of earlier studies to offer notable neuroprotection against acute stroke via the induction of inflammatory responses, suppression of neuronal apoptosis, and stimulation of angiogenesis [95,96]. In this regard, preconditioning exercise has been shown by Otsuka et al. [97] to minimize neurological impairments; alleviate sensorimotor dysfunction and consciousness disturbance; and reduce inflammation, oxidative stress, and neuronal death. The 14-3-3γ/*p*-β-catenin Ser37/Bax/caspase-3 anti-apoptotic pathway and the amplified activated Nrf2/HO-1 anti-oxidative stress pathway are responsible for these effects. Following subarachnoid hemorrhage (SAH), rats’ basilar arteries express Nrf2 more frequently [98]. Furthermore, according to a prior study [99], SAH tests in mice employing a Nrf2 deletion model revealed greater brain edema and neuronal death as well as lower neurological scores in these mice as compared to wild-type animals. Nrf2 binds to ARE sequences to regulate a variety of protective factors. Furthermore, oxidative stress, apoptosis, autophagy, inflammation, and mitochondrial function are all significantly impacted by the Nrf2/ARE pathway [100]. Exercise of a specific intensity and duration is necessary for Nrf2 expression in the brain. Through the activated Nrf2/HO-1 pathway, six weeks of moderate-intensity exercise protects against experimental 6-hydroxydopamine-induced hemiparkinsonism [101].

The results of this investigation showed that ovariectomy reduces brain BDNF. Exercise, IF, and their combination lessened the negative effects of ovariectomy on BDNF levels. The combined strategy had no more impact than either exercise or IF by itself. These findings imply that exercise and IF may be able to counteract the detrimental effects of ovariectomy on BDNF levels in an experimental menopausal rat model. Numerous studies have demonstrated that the hormonal state affects the production of BDNF and/or its receptor; hence, there may be a biological substrate for the gonadal hormone regulation of this neurotrophin [102]. The persistent absence of estrogen decreased the amounts of BDNF mRNA in various cortical and hippocampus areas. If the estrogen replacement was started reasonably quickly after the OVX (within 3 weeks) but not after a prolonged period of estrogen deprivation, BDNF mRNA levels in some of the hippocampus regions could be restored over several weeks (5–25 weeks) [103]. The hippocampal BDNF receptors and BDNF levels are enhanced by systemic estrogen treatment [104]. Estrogens and BDNF both improve dendritic spines in the prefrontal cortex and hippocampal regions as well as cognitive performance [102]. Estrogens may therefore improve cognitive performance and brain morphology through BDNF. Additionally, new research suggests that BDNF and estrogens may cooperate to enhance memory and learning. In mature ovariectomized female rats, the replacement of estrogens boosted the expression of BDNF in various brain areas, including the hippocampus [105]. In our investigation, IF was linked to higher amounts of BDNF in the brains of rats that had had their ovaries removed. Through an increase in blood estrogen levels, exercise also helped ovariectomized rats exhibit less depressive behaviour [106]. Additionally, it has been demonstrated that, through promoting cell proliferation and inhibiting apoptosis, combined exercise improves the cognitive impairment brought on by ovariectomy [107]. According to Sartori et al. [108] and Rocha-Gomes et al. [109], exercise is linked to higher mBDNF expression and signaling and is good for hippocampus functioning. Furthermore, exercise has a part in protecting and enhancing the hippocampal tissue in neurodegenerative illnesses [110]. According to Jin et al. [111], in a rat model of OVX, exercise enhanced cognitive skills. According to Park et al. [112], rats that were given OVX and regularly treadmill exercised afterward had better BDNF pathways in the hippocampus, with levels of mBDNF, TrkB, and tPA rising and proBDNF, p75NTR, and JNK falling. The findings indicated that, by raising mBDNF synthesis and activating tPA, exercise may inhibit the proapoptotic response of the proBDNF-p75NTR pathway. Therefore, in the brain of an OVX rat, exercise may improve neuronal functions. Additionally, our findings demonstrated that fasting significantly increased BDNF in OVX animals. Similar effects on BDNF levels have been found in other investigations. In the rat model of type 2 diabetes mellitus [83] and in normal mice [113], rats undergo extended fasting. Furthermore, Pan et al. found that calorie restriction raised levels of brain-derived neurotrophic factor (BDNF) in the hippocampus and cortex, while lowering BDNF expression in the areas of the hindbrain and hypothalamus that control feeding and metabolic efficiency [114]. Additionally, research revealed that following a 48 h fast BDNF protein levels dropped and then rose following refeeding [115]. These findings imply that different brain regions respond differently to calorie restriction or fasting in terms of BDNF levels.

## 5. Conclusions

These results suggest that ovariectomy can impair rat neuronal function and regular treadmill exercise and intermittent fasting seem to protect against ovariectomy-induced neuronal impairment through the inhibition of oxidative stress and neuroinflammation and increased BDNF levels in the brain cortex. Combining regular exercise and intermittent fasting did not provide additional benefits compared to either treatment alone. This is the first study to evaluate the combined effect of regular exercise and intermittent fasting in estrogen deficiency induced by ovariectomy. Clinical studies are warranted to confirm the beneficial effects of regular exercise and intermittent fasting in postmenopausal women.

## Figures and Tables

**Table 1:** Primer sequences of genes analyzed in real-time PCR.

Name	Accession Number	Forward Primer (5′-3′)	Reverse Primer (5′-3′)
GAPDH	NM_017008.4	CTCTCTGCTCCTCCCTGTTC	TACGGCCAAATCCGTTCACA
SOD2	NM_017051.2	CGGGGGCCATATCAATCACA	GCCTCCAGCAACTCTCCTTT
SOD1	NM_017050.1	TGGTGGTCCACGAGAAACAA	GCAATCCCAATCACACCACAA
GPx1	NM_030826.4	CCTGGTATCTGGGCTTGGTG	TTAGGCGTAAAGGCATCGGG
GPx4	NM_001039849.3	AAGTCCTAGGAAGCGCCCA	GGGTTGAAAGGCTCGGGAAT
BDNF	NM_001270630.1	AATAATGTCTGACCCCAGTGCC	ATTGTTGTCACGCTCCTGGT

## Data Availability

All relevant data are within this paper.
